# Therapists’ Emotional Responses in Individual Therapy with Depressed Adolescents: An Evaluation of the Data Structure of the Feeling-Word Checklist—28

**DOI:** 10.3390/ijerph19159496

**Published:** 2022-08-02

**Authors:** Pernille Brøsholen, Randi Ulberg, Hanne-Sofie Johnsen Dahl, Agneta Thorén

**Affiliations:** 1 Institute of Clinical Medicine, University of Oslo, P.O. Box 1039, Blindern, 0315 Oslo, Norway; randi.ulberg@medisin.uio.no; 2 Research Unit, Division of Mental Health and Addiction, Vestfold Hospital Trust, P.O. Box 2169, 3125 Tønsberg, Norway; h.s.j.dahl@psykologi.uio.no; 3 Department of Psychiatry, Diakonhjemmet Hospital, P.O. Box 85, Vinderen, 0319 Oslo, Norway; 4 Department of Psychology, Faculty of Social Sciences, University of Oslo, P.O. Box 1094, Blindern, 0317 Oslo, Norway; 5 The Erica Foundation, Odengatan 9, P.O. Box 114 24, 911424 Stockholm, Sweden; agneta.thoren@ericastiftelsen.se

**Keywords:** countertransference, adolescents, feeling word checklist, factor analysis, principal component analysis, major depressive disorder, psychometrics

## Abstract

Countertransference (CT) responses during therapy sessions can be understood as the therapist’s emotional reactions towards the patient. Within adolescents’ psychotherapy, little is known about the effects of the therapists’ feelings on treatment outcome. The Feeling-Word Checklist—28 (FWC-28) is a self-report questionnaire designed to evaluate the therapist’s in-session feelings during therapy with younger patients. The aim of the study was to evaluate the psychometric properties of the clinician-rated FWC-28 and explore the associations between the CT-subscales and therapeutic alliance. Data were collected from a randomized controlled trial in which 11 therapists specialized in child and adolescent psychotherapy treated 16- to 18-year-old patients (*n* = 62) with major depressive disorder in outpatient clinics. The patients received psychodynamic psychotherapy treatment over 28 sessions. Therapists rated their emotional responses towards their patients on FWC-28 after sessions 3, 12, 20, and 28. Principal component analysis (PCA) with oblique rotation was performed to find clinically meaningful subscales of the FWC-28. PCA revealed four clinically meaningful components termed as follows: inadequate, confident, motherly, and disengaged. The psychometric properties of the FWC and the reliability of the CT subscales measured with Cronbach’s alpha were acceptable. The therapist-reported alliance showed significant and clinically meaningful correlations with all CT-subscales. Our findings indicate that the checklist is adequate for clinical practice and countertransference research in adolescents’ psychotherapy.

## 1. Introduction

All health professionals experience various emotions when encountering patients regardless of theoretical background or amount of experience and competence [1]. Several studies have shown that the therapeutic relationship [2,3,4] as well as the therapist’s management of their own feelings [5] are important predictors of patient outcome [1]. Such findings have led to an increased interest in mapping various aspects of the therapeutic relationship and understanding how therapists’ emotional reactions can affect the psychotherapy process [6].

Depression is one of the leading causes of illness and disability among adolescents, as it causes mental suffering and prevents development and maintenance of social and emotional skills [7]. Early interventions can prevent severe outcomes and progression of primary disorders [8]. However, some adolescents with depression do not respond to psychotherapy treatment. Recognizing the nature of emotions in the patient–therapist interaction and tailoring more personalized treatment for each specific patient may be necessary to reduce non-responsiveness and drop-out rates, as it is clear that: “one treatment does not fit all” [9].

### 1.1. Research on Adolescents Psychotherapy

Treating adolescents with depression could be a complex task. Adolescence is a phase of life colored by identity seeking, fluctuating emotions, insecurity, and physical, intellectual, emotional, and social developmental changes [10]. The therapist must have the capacity for adaption and flexibility and should be prepared for rapid shifts in the adolescent’s mood and behavior. Adolescents can evoke strong feelings by nature, and they tend to induce reactions in their therapists that are unique in terms of their affective value and intensity [11]. The therapists’ authority and professional competence can often be challenged by adolescents, which can further lead to feelings of discomfort and inadequacy in the therapist [11]. Adolescents are therefore of particular interest for studying therapists’ emotional responses.

### 1.2. Countertransference

The therapist’s emotional reaction is understood to be a part of the countertransference response. Countertransference may be defined as the therapist’s experiences and emotional reactions towards the patient, both conscious and unconscious [12]. It was originally defined by Freud as a phenomenon in the context of the psychoanalytic approach, suggesting that the patient’s influence on the therapist’s unconscious feelings could disturb the therapist. Early perspectives described countertransference phenomena as an obstacle to treatment: therapists should stay objective and neutral, not allowing any personal feelings and inner conflicts to interfere with therapy [13]. Over time, the concept broadened with the desire to establish a greater understanding of the phenomena [11]. Paula Heimann was one of the first psychiatrists to define countertransference with a positive value [11]. She posited that the therapist’s countertransference feelings could be a reflection of the patient’s personality and as such give valuable information about the patient. Heimann proposed that the emotional response experienced by the therapist towards their patient should be regarded as an important tool for therapeutic work and a greater understanding of the relationship between the patient and therapist [14]. The broadened view was later referred to as the totalistic view [15] and shifted the view of countertransference from being an obstacle to treatment to a potential source of understanding of patients’ experiences. The totalistic approach suggested, among other things, that the therapists’ emotional reactions toward their patients are influenced by the past and present, unconscious and conscious feelings, as well as their beliefs, attitudes, thoughts, and motivations [15].

It has been argued that the totalistic definition of countertransference runs the risk of diverting attention away from the therapist and the influence of their own personal conflicts [16]. The integrative conception [17] locates the source of therapist’s reactions to the patients as residing within the therapist. Tishby and colleagues [18] examined the origin of therapist’s reactions towards adolescents patients, mainly their interpersonal patterns with parents, by adopting the integrative definition of countertransference. They measured countertransference reactions in therapists towards their adolescent patients with the CCRT method [19] and found it to be a very useful method for capturing themes from the therapist’s past that reappear in the relationship with young patients, such as repetitiveness of their own parent themes in the narratives about the patients.

Despite the disagreements regarding the specific definition of countertransference, the growing body of empirical literature suggests that countertransference is considered as a valuable source of information about the patient–therapist relationship and potentially a useful tool for practitioners across a wide array of backgrounds [20,21]. Clinicians’ reactions towards their patients could potentially have diagnostic and therapeutic relevance and, if appropriately handled, can facilitate alliance and improve the treatment process [14,15,22].

### 1.3. The Feeling Word Checklist

The Feeling-Word Checklist (FWC) is a self-report questionnaire comprised of different feeling words where therapists rate their emotional response towards a patient. There are various versions of the FWC, and it is one of the most used questionnaires in research on therapists’ feelings [23]. Whyte and colleagues [24] constructed the first version of the FWC, applied in empirical research, as a 30-item scale with yes or no options. Since then, different versions with various items ranging from 12 to 58 feeling words, mostly with Likert scales, have been developed [6,25,26,27,28]. Nearly all studies on FWC have been completed with clinician ratings but in different clinical settings, such as inpatient [27,29,30] or individual psychotherapies [25,26,31,32].

***Feeling-word checklist in individual therapy.*** Despite the amount of theoretical literature and focus on the therapist’s feelings in therapy, only a few studies to date have examined the therapists’ feelings with the FWC in individual therapy [25,26,31,32]. Dahl and colleagues [25] investigated therapists’ emotional reactions towards adult patients in one year of individual therapy. Using a feeling-word checklist consisting of 58 items, principal component analysis (PCA) revealed four components named confident, inadequate, parental, and disengaged, which seemed to capture clinically meaningful aspects of the therapeutic dyad with acceptable psychometric properties. Breivik and colleagues [33] found similar factors named inadequate, idealized, and confident when evaluating a 12-item version of the FWC (FWC-BV) and concluded that the FWC-BV seemed satisfactory for clinical purpose and use in further research.

***Feeling-word checklist in adolescents.*** Little empirical attention has been paid to the clinician responses towards adolescent patients in treatment, and only a few studies have explored the therapist’s subjective experiences [8,26,34,35,36]. Ulberg and colleagues [26] studied the emotional responses of 41 clinicians towards their adolescent patients (*n* = 410) with the FWC-24. They found four subscales with adequate psychometric properties termed: confident, inadequate, neutral, and disengaged. The subscales seemed to represent different feeling aspects and partly overlapped with earlier research in adults. The findings demonstrated that the confident CT subscale was positively associated with clinician age, more clinical supervision, and more practical experience. In a related study, Tanzilli and colleagues [36] used the Therapist Response Questionnaire for Adolescents [35] and investigated the relationship between therapists’ emotional response towards their adolescent patients during treatment and the patients’ psychological functioning. Their findings showed that high levels of psychological functioning were positively related to a positive countertransference response (warm/attuned) and negatively related to a negative CT response (disengaged/hopeless, angry/criticized, overinvolved/worried) [36].

***Feeling-word checklist and therapeutic alliance.*** There are only a few studies that have investigated the relationship between therapeutic alliance and countertransference. A few studies have reported both positive correlations between therapeutic alliance and positive countertransference feelings and negative correlations between therapeutic alliance and negative countertransference feelings [25,26]. Dahl and colleagues [25] found a strong negative correlation between therapist-rated alliance and therapist reports of feeling “disengaged”. There are no studies to our knowledge on adolescents with MDD that have examined the correlations between therapeutic alliance as reported by both patient and therapist and the countertransference feelings of the therapist. Ulberg and colleagues, however, studied therapists’ emotions when treating young adults with a broad range of clinical problems and found that the therapist’s feelings of “confidence” were positively associated with therapist-rated alliance, while the feelings of being “inadequate” or “disengaged” were negatively associated with alliance [26].

Various statistical methods have been used to analyze and evaluate FWC, and different components/factors have been described. The diversity of components/factors could be explained by the use of various FWC versions and different scale formats. Previous studies have included therapists from different professions and various patient populations, and the sample sizes in the different studies also vary. There is no consensus about which FWC version best captures therapists’ feelings. Nevertheless, a consistent finding across various versions of the FWC is that at least one factor reflects positive feelings, and at least one factor reflects negative feelings [6]. Recent studies have also shown that self-reports can measure the therapists’ feelings in clinically sound ways by showing meaningful correlations with patient and therapist characteristics and various alliance measures [6,25,37,38,39].

The Feeling-Word Checklist—28 has never been applied in any research in individual therapy, and the scientific exploration on the effect of the therapists’ feelings in therapy with adolescents is sparse. In the present study, we examined therapists’ emotional responses towards their patients during therapy sessions—more specifically the feelings evoked when treating adolescent patients with major depressive disorder (MDD). This study integrates numerous possible sources of feelings evoked in the therapist, including patient dimensions such as personality, history, and pathology; the therapy itself; and therapist dimensions such as their own personality, history, and experience.

## 2. Aims and Objectives

The main aim of the current study was to explore the underlying structure of the FWC-28 when applied in adolescent psychodynamic therapy for major depression. A secondary aim was to evaluate the psychometric properties of the FWC-28 and validate the CT-subscales by exploring the relationship between the components and alliance-measures rated by both patient and therapist.

### Research Questions

1. How many clinically meaningful subscales do the items of the FWC-28 constitute in psychodynamic psychotherapy with 62 adolescent patients diagnosed with major depression, and what are their psychometric properties?
2. Is there a significant relationship between the CT subscales of the FWC-28 and alliance measures as reported by both patient and therapist in psychodynamic therapy with depressed adolescents?

## 3. Material and Methods

The data were collected as part of the First Experimental Study of Transference Work in Teenagers (FEST-IT); a multicenter observer- and patient-blind, randomized controlled component trial [40]. From February 2012 to September 2017, adolescent patients suffering from major depression were randomly allocated to one of two treatment groups. Patients were assigned to one out of eleven specifically trained therapists who treated patients in both groups. To assess and capture the therapists’ CT feelings, a feeling-word checklist with 28 feeling words (FWC-28) was applied. 

### 3.1. Ethical Approvals

The Central Norway regional Ethics Health Committee approved the study protocol (REK: 2011/1424 FEST-IT). The specifications for the present project were accepted by REK as a project amendment (1 March 2021). FEST-IT is registered in ClinicalTrials.gov (accessed on 18 November 2021): NCT01531101. The data collected for this project are stored at the services for sensitive data at the University of Oslo (TSD) [8]. Studying adolescents in psychotherapy can be very sensitive and require strict confidentiality and a focus on the integrity of the individuals. According to Norwegian legislation, ethical consent for participation in research may be given by the participant him-/herself from the age of 16. The participants were, however, encouraged to discuss participation with their guardians.

### 3.2. Treatment Conditions

Patients were randomized to one of two treatment groups, in which short-term psychoanalytic psychotherapy was used as the manual for the treatment [41]. Patients were treated weekly with 45-minute sessions over 28 weeks. Patients in the two treatment groups received either a moderate level of transference intervention (*n* = 39) or no transference intervention (*n* = 31). Transference work is a specific technique in psychodynamic therapy in which there is an active focus on the ongoing patient–therapist relationship during the therapy sessions [8]. In the transference group, therapists encouraged the patients to explore their feelings and thoughts about the therapist and the therapy. Further, they explored repetitive patterns of reactions and actions emerging during the sessions in relation to the therapist. These interventions were offered to a moderate level (1–3 times per session) [8]. The mean number of sessions were 18.6 (SD = 8.6) in the transference work group and 18.0 (SD = 10.9) in the non-transference work group.

### 3.3. Participants

***Therapists*.** The 11 therapists (*n* female = 5, *n* male = 6) were all specialized in psychiatry or clinical psychology. The therapists had different amounts of experience, some with minimum requirements of training and others with more than 30 years of experience as psychodynamic psychotherapists. All the therapists had at least 2 years of formal education in psychodynamic psychotherapy. The therapists were trained in a one-year training program to provide dynamic psychotherapy with moderate frequency of transference work and without transference work. Peer supervisions were offered to the therapists during the trial period in order to maintain adherence and quality of the therapies [8,40].

***Patients*.** The patients included were adolescents aged 16 to 18 (mean = 17.3, SD = 0.7) years old, with current unipolar major depressive disorder (MDD) according to Diagnostic and Statistical Manual of Mental Disorders, Fourth Edition (DSM-IV; American Psychiatric Association, 2000). The patients were included from two areas in Norway: the mainly urban areas in and around Oslo populated by approximately one million people and the mixed urban and rural areas in Vestfold County, with a population of about 250,000 people. There was a majority of female participants (*n* female = 51, *n* male = 11). The patients were treated in child and adolescent psychiatric outpatient clinics. Adolescents with generalized learning difficulties, pervasive developmental disorder, psychosis, or substance addiction were excluded. To ensure external validity in the sample, no other patients were excluded, and the comorbidities listed in Table 1 were frequent, as expected. Only two patients received antidepressant medication. All patients participated in a one-year follow-up after treatment termination [8,40].

Seventy patients were initially recruited to FEST-IT, and one patient withdrew from the study. The FWC-28 was not completed by the therapist for seven of the patients at any time. Thus, the final patient and therapist sample included in the current study consisted of 62 patients and 11 therapists.

### 3.4. Recruitment, Randomization, and Masking

Therapists invited the patient to sign a consent form if they met the inclusion criteria, whereby written informed consent was obtained from all patients. Before randomization, each patient was interviewed using the M.I.N.I 6.0.0, which is a structured interview used to diagnose psychiatric symptoms according to DSM-IV criteria, and the SIDP-IV structured interview for DSM-IV personality disorders [42]. They also underwent a 1-h psychodynamic interview (PI) modified after Malan [43] and Sifneos [44]. If the patient met the criteria for major depression on M.I.N.I, therapists would contact one of the researchers in the study. Following baseline assessments, the patients were assigned a trial ID. Patients were randomized to one of two treatment groups by a randomization officer with no other connection with the evaluators, therapists, study coordinators, or researchers [8,40].

### 3.5. Measures

***Feeling-Word Checklist.*** The Feeling-Word Checklist—28 is a modified version particularly designed to assess CT feelings experienced by therapists when treating younger patients. The therapists were asked to rate to what degree they experienced the 28 feeling states towards their patients, such as happy, irritated, warm, bored, etc., on a 4-point Likert scale ranging from “nothing” to “very much”. The questionnaire was administered after the 3rd, 12th, 20th, and 28th sessions, and the number of FWC-28 questionnaires collected were 53, 48, 36, and 36, respectively, yielding a total of 174 questionnaires. It took about 5 min to complete the questionnaire. FWC-28 was designed based on the FWC-24 [32,45], which has been applied in previous research on CT responses. The checklist was expanded by the research team with four feeling words, namely motherly, affectionate, dominant, and important, which were found to be crucial in Dahl and colleagues’ study [25,46] on the therapist countertransference feelings with adult patients. The feeling words constituted Dahl and colleagues’ “parental” subscale, which showed associations with long-term specific effects. Therefore, we wanted to include the specific items in the present study, as they were assumed to be important in therapy with adolescents. The four feeling words were added to the checklist after the treatment had started and were included in 123 out a total of 174 questionnaires. The items of the FWC-28 are presented in Table 2.

***Alliance measures.*** The therapist–patient alliance captures the quality of the working relationship in therapy and is seen as an important aspect of every effective psychotherapy. Patients and therapists filled out the Working Alliance Inventory—Short Version (WAI-SR) [47] in sessions 3, 12, 20, and 28. WAI-SR is a 12-item questionnaire where therapists and patients are asked to judge different aspects of their relationship on a Likert scale from 1 = “never” to 7 = “always”. Patient- and therapist-assessed WAI is termed WAI-P and WAI-T, respectively. In addition to WAI, the therapist’s alliance was also measured with the visual analogue scale (VAS) [48]. The VAS scale asks the therapists to mark a point on a 10 cm line to judge to what degree they “like to treat this patient”. To explore the relationship between alliance and CT subscales and patients variables, we included data on WAI and VAS scores from sessions with the 62 patients across the four measurement points as separate measurements and the average scores of the four measurement points. WAI-P, WAI-T, and VAS scores were available for 159, 172, and 168 sessions, respectively, out of the total of 174 possible correlations with CT subscales.

## 4. Statistical Analysis

The analyses were performed in IBM SPSS-28, and calculations are from the analysis of data collected from sessions 3, 12, 20, and 28 for all patients as separate measurements. To explore the structure of the FWC-28, a principal component analysis (PCA) was performed on data from all sessions. PCA is a data reduction technique in which the number of variables is reduced into a smaller set of components that can account for most of the variance in the observed variables. We rotated the components with oblique promax rotation due to the a priori expectation of correlations between the components from the FWC-28. The purpose of rotation is to identify groups of variables that correlate highly with each other and little with other variables outside that group [49].

Since multiple questionnaires were filled out for each patient, we conducted a PCA on aggregated data comprising the average scores of the individual feeling words across the four measurement points for all patients (*n* = 62) conducted by the same therapist to validate whether the same factor structure was obtained.

To pick the number of components to rotate, we used the Kaiser eigenvalue > 1, a scree plot, variance explained, and clinical interpretability of the components. We wanted to keep the items that loaded highly with only one subscale and remove items that loaded highly with more than one subscale. Dahl and colleagues’ [25] specific procedure was followed to remove items that did not follow suit. Items from the initial pattern matrix were excluded if they loaded less than 0.35 on all components and above 0.30 on more than one factor. We reran the analysis by extracting the number of components to yield a final solution.

The items of the FWC-28 were expected to be correlated with each other, and thus, we decided to calculate the Cronbach’s alpha to assess the scale reliability and explore the internal consistencies of the subscales. To examine correlations between the CT subscales and the correlations between the CT subscales and alliance measures, Pearson product moment coefficients (r) were used. The significance level was set to 0.05.

## 5. Results

### Principal Component Analysis

To verify that our data were suited for a principal component analysis (PCA), the Kaiser–Meyer–Olkin measure was calculated (KMO > 8.1). The Bartletts test of sphericity x^2^ (378) = 1553.529, *p* < 0.001, indicated that the correlations between the items were sufficient to conduct a PCA.

The PCA conducted from all data revealed four CT subscales that provided the best fit for the data. The four subscales explain > 52% of the variance. The subscales were named inadequate, confident, motherly, and disengaged, including five items each. The pattern matrix shows the component loadings—the regression coefficients—of the observed variables, expressed as a function of the components as displayed in Table 2. The first subscale, inadequate, explains 25.7% of the variance and includes feeling words that are expected to appear when the therapist is feeling tense or uncomfortable. The confident subscale explains 12.2% of the variance and includes feeling words that are likely to occur when the therapist is experiencing feelings of satisfaction and being helpful. The motherly subscale explains 8.4% of the variance and involves items illustrating feelings of being affectionate, warm, important, and in touch with the patient. The disengaged subscale explains 6.4% of the variance and represents feelings of being cold, indifferent, and uninterested. Similar subscales emerged when we ran the PCA on the aggregated data, which could explain >61% of the variance. The CT subscales found contain 20 of the original 28 items of the FWC-28.

## 6. Validation and Evaluation

The correlations between the subscale scores and the factor scores were >0.91 on all components, indicating that the subscale scores might substitute the factor scores without loss of information [25]. The internal consistency, calculated from all data, measured with Cronbach’s alpha were acceptable for three of the components and questionable for one. The alpha was 0.71 for inadequate, 0.81 for confident, 0.76 for motherly, and 0.67 for disengaged. The Cronbach’s alpha does not test whether the individual items are really influenced by only one or by several latent variables, and the coefficient must therefore be interpreted with caution [50].

Intercorrelations between the four CT subscales with data from all sessions (*n* = 174) and aggregated data (*n* = 62) were assessed using Pearson’s correlations (r). There was a moderate correlation between some of the subscales, as shown in Table 3. On data from all treatments, the subscales confident and motherly revealed a significant correlation (*r* = 0.493, *p* < 0.001). This correlation was also found to be moderate on aggregated data (*r* = 0.511, *p* < 0.001). There was a weak but also significant negative correlation between inadequate and confident (*r* = −0.215, *p* = 0.004) on data from all treatments, which is reasonable due to the contradictive feelings underlying the two subscales. There was, however, no correlation between inadequate and confident in the aggregated data. There was a weak, significant correlation between disengaged and Inadequate on data from all sessions (*r* = 0.222, *p* = 0.003) and the aggregated data (*r* = 0.265, *p* = 0.037), which also seems clinically meaningful. There was no significant correlation between motherly and disengaged, motherly and inadequate, and confident and disengaged. The moderate intercorrelations between the subscales might indicate the presence of a common positive–negative feeling factor confounding the current CT subscales.

Table 4 summarizes descriptive statistics of CT subscales from all sessions. The mean value over all treatments is highest on the motherly subscale. The inadequate CT subscale has the lowest mean, which overlaps with Dahl and colleagues’ previous findings [25].

The alliance measured with Working Alliance Inventory (WAI) was on average rated quite high by the patients; WAI-Patient (WAI-P) mean = 5.41 (*SD* = 0.88) and slightly lower by the therapists: WAI-Therapist (WAI-T) mean = 4.9 (*SD* = 0.88). The therapist-rated VAS was rated with a mean of 6.8 (*SD* = 2.0). There was a strong correlation between WAI-T and VAS on all data (r = 678, *p* ≤ 0.001) and aggregated data (r = 0.77, *p* ≤ 0.001). We found a weak but significant correlation (*r* = 0.223, *p* = 0.004) between the WAI-P and WAI-T measures, conducted from all data.

Correlations between the four CT subscales and the alliance measures, WAI and VAS, were assessed with data from all sessions and aggregated data. Table 5 shows the correlations between therapist-rated WAI (WAI-T) and the CT subscales. All the subscales showed a significant correlation with WAI-T. There was a negative correlation with the inadequate subscale on all data (r = −0.322, *p* < 0.001) and on aggregated data (*r* = −0.352, *p* < 0.001) and the disengaged subscale on all data (*r* = −0.286, *p* < 0.001) and aggregated data (*r* = −0.347, *p* < 0.001). WAI-T revealed a positive moderate correlation with the confident CT-subscale on all data (*r* = 0.502, *p* < 0.001) and aggregated data (*r* = 0.598, *p* < 0.001) as well as with the motherly CT subscale on all data (*r* = 0.435, *p* < 0.001) and aggregated data (*r* = 0.594, *p* < 0.001). VAS scores on data from all sessions and the aggregated data showed a significant correlation with all the subscales as shown in Table 5.

Table 5 also shows correlations between WAI-P and the CT subscales. There is a significant positive correlation between WAI-P and the confident CT subscale on all data (*r* = 0.186, *p* = 0.019) and aggregated data (*r* = 0.345, *p* = 0.008) but no significant correlation with the remaining CT subscales.

## 7. Discussion

The primary aim of the study was to explore the factor structure of the Feeling-Word Checklist—28 and evaluate its psychometric properties. Using a principal component analysis (PCA), we found 20 items that constituted four clinical, meaningful, and psychometrical acceptable CT subscales named inadequate, confident, motherly, and disengaged. A PCA is not strictly a factor analysis (FA), but the results and methods are so similar that it is often misunderstood as a type of factor analysis. However, mutual factors or components have been observed in empirical studies with various populations such as adult and adolescent patients with a variety of mental disorders, using both proper FA and PCA. FA and PCA has been conducted in outpatient and inpatient settings, and the sample sizes vary [6,25,26,27,30,33].

In line with the current study, previous related work with data conducted from individual therapy, [6,25,26,31] have found four components/factors, similar to our present study. Holmqvist and colleagues [31] used a FWC consisting of 48 items (FWC-48) and found four factors to be suggested: positive, negative, distant, and dejected. Their negative and dejected CT subscale shares similarities with our inadequate CT subscale, and their distant subscale consists of items that conceptually overlap with our disengaged CT subscale. Further, Dahl and colleagues [25] found similar components in their study with FWC-58 when used with adult patients. The subscales were labelled confident, inadequate, disengaged, and parental. Three of our CT subscales were labelled the same as theirs, and all four components conceptually overlap with the subscales found in the present study. Unlike our motherly subscale, their parental subscale includes the feeling word “dominating”, which has led to different labelling of subscales.

Lindquist and colleagues [6] used a version with 24 feeling words (FWC-24) with a sample consisting of children, adolescents, and young adults. They found four subscales named engaged, inadequate, relaxed, and moved. The engaged subscale shares similarities with our confident CT subscale, which describes being energetic, enthusiastic, confident, and happy. Further, the inadequate subscale shows similarities with both our disengaged and inadequate CT subscale. Dahl and colleagues [25] suggested that some therapists may yield a disengaged stance as a self-protective act against aggressive feelings, potentially explaining why our inadequate and disengaged subscale both conceptually overlap with the inadequate subscale found by Lindquist and colleagues [6]. Ulberg and colleagues [26] found four components with their FWC-24 named confident, inadequate, disengaged, and neutral. Their inadequate and confident subscale includes feeling words that conceptually overlap with our confident and inadequate subscales. Additionally, their disengaged subscale somewhat overlaps with our disengaged subscale except that our disengaged subscale also includes the feeling word “neutral”: a feeling word that Ulberg and colleagues found to be an own subscale.

The concept of “neutrality” originated within psychoanalysis as the most operative and practical therapist standpoint [51] and has been a concept of great discussion over the past decades. The neutral subscale found by Ulberg and colleagues [26] showed a positive correlation with the therapists’ age, years of clinical experience, education, and supervision [26]. They proposed that the neutral subscale might mirror a pragmatic stance in more experienced, highly educated, and older therapists. Other previous studies have shown that the feeling words “neutral” and “objective” covary among positive countertransference feelings [25,26]. In our analysis, the feeling word “neutral” had a high factor loading with the disengaged subscale and was associated with other negative feelings such as feeling aloof, cold, uninterested, and tired. Based on the varying findings regarding the concept of neutrality, it seems that neutrality can be both positively and negatively valued, reflecting a shift in the therapeutic stance over the years. Due to the different understandings of the term “neutral”, it can be somewhat problematic to use the term as an item in a feeling-word checklist.

The mean CT subscale scores range from 0.54 to 1.65, in which motherly and confident are assigned the highest scores. The scores are similar to those observed in other studies of different versions of the FWC, which captured quite low scores [6,25,26,33]. Breivik and colleagues [33] studied the FWC used in contexts of patients with personality disorders and found that the confident subscale had the highest mean value, something they found somewhat surprising. They suggested that confident had the highest score due to the therapists being highly experienced and that regular supervision and focus on CT has been a central part of the therapists’ work. They found that lower levels of inadequate feelings were associated with more supervision [33]. The findings of Ulberg and colleagues [26] support this idea, where the confident subscale was positively associated with more experienced therapists as well as an increased level of supervision. This might also be the case in our findings; the high mean scores for the motherly and confident subscales could be explained by the therapist’s high level of experience and regular supervision on how to manage CT feelings during therapy.

Regarding the correlations between the alliance measures and CT subscales, we found systematic correlations with meaningful values. The inadequate and disengaged subscales show a moderate negative correlation with alliance, and the confident and motherly subscales show a moderate positive correlation with alliance. These correlations show that, not surprisingly, the therapists feelings affect how they experience the alliance and support the conceptual meaningfulness of the subscales. We also found a strong correlation between the alliance measures applied in the present study: WAI-T and VAS. Ulberg and colleagues [26] found similar correlations in which the therapist-rated visual-analogue scale (VAS) showed a positive correlation with the confident CT subscale and a negative correlation with the inadequate and disengaged CT subscales. The correlations seem to have clinical value, as it is plausible that positive CT feelings reported by the therapists will have a positive correlation with good therapist-reported working alliance and vice versa. We have only found a few studies that have explored the relationship between therapists’ feelings and the alliance [25,26,52,53]. Among other things, Tishby and Wiseman [53] found that negative CT patterns were associated with more ruptures and less resolution with the patients.

As mentioned, the CT subscales in the current study contain five items each. Although the four components only contain 20/28 of the original FWC-items, we believe that the eight remaining feeling words are likely to be triggered in the therapist when treating adolescent patients with major depression or patients with other psychiatric disorders. By excluding the eight feeling words, we might risk losing an important spectrum of feelings that the therapist may experience. Thus, we believe that the FWC-28 should keep all the original 28 items in future research.

## 8. Limitations

The interdependencies in our data represent an inescapable limitation and a noteworthy weakness of the present study. There is a significant interdependency on both the patient and therapist level because of the repeated measures on the same patients over the treatment course by the same therapists. The first PCA we performed on all the FWC-28 questionnaires (*n* = 172) gave a ratio of subjects to items = 6:1, which is argued to be an acceptable ratio for collecting stable estimates. However, the data within each case are not independent because of the repeated measures. We found the average scores for each patient over the total course of treatment and ran a PCA on this aggregated data (*n* = 62) to further investigate whether it proposed the same structure. This will, however, introduce another problem regarding the sample size. With the aggregated data, we get *n* = 62, which is quite low for running a PCA, and the ratio of subjects to items is approximately around 2:1. A subject-to-item ratio of at least 5:1 has been suggested as a minimum when running a PCA [54], and therefore, there is a risk of uncertain estimates and unstable data structure.

Calculating the average scores collected from several sessions with the same patients makes it challenging to generalize our findings to situations where the instruments have been answered once, complicating the investigation of the validity of our findings. However, we found similar factor structures from the analysis on all data and aggregated data as well as previous studies examining different feeling-word checklists.

Each patient–therapist dyad is unique. These meetings will nevertheless be characterized by the therapist’s personality, history, and experience, making this a significant dependent component in our analysis. The therapists in the present study had different amounts of experience, some with minimum requirements of training and others with more than 30 years of experience as psychodynamic psychotherapists. The variation in the amount of experience may have influenced the results in the present study. For example, Ulberg and colleagues [26] revealed positive correlations between their confident subscale and the therapist’s age, length of education, supervision, and years of working in child and adolescent psychiatry. Unfortunately, the present study lacks detailed information about the therapists, and such correlations could not be examined. Furthermore, the therapist sample size (*n* = 11) is very small, and we can therefore not claim that the therapists constitute a representative sample. Research on therapists’ emotions without interdependencies on both patient and therapist level and with a greater therapist sample size is needed to confirm our findings.

In the present study, the framework for understanding countertransference reactions is the psychodynamic treatment situation. One can argue that therapists specialized in psychodynamic psychotherapy are quite aware of their feelings during therapy, as they often have received a great amount of supervision. The therapists in the present study attended supervision during the study period, which might have affected their awareness of their countertransference reactions. However, Betan and colleagues [37] found similar patterns of countertransference response in treatment regardless of the therapist’s theoretical background, supervision, or whether they believed in the concept of countertransference. Although countertransference emerged as a concept in psychoanalytic theory, findings suggest that therapists of all theoretical orientations can make use of information provided in the context of the therapeutic relationship, including their feelings towards their patients [37].

It is difficult to compare studies that have examined therapists’ feelings with a feeling-word checklist (FWC), partly because different versions of FWC’s have been applied. Furthermore, therapists’ feelings have been examined when treating different patient populations. This can generate various findings, as different patient characteristics and age groups are likely to evoke a variety of feelings. It is important to note that our sample is not just young people, but they also suffer from major depression. Depressive states in patients may easily influence therapists’ feelings. Furthermore, no adolescent patients from minority populations were referred, and there was a majority of female participants in the current study. Hence, there are generalizability issues.

## 9. Conclusions

In the present study a principal component analysis revealed four subscales of the Feeling-Word Checklist—28 named inadequate, confident, motherly, and disengaged. The subscales seemed clinically meaningful and, to some extent, corresponding with subscales found in other previous studies. The psychometric properties of the FWC-28 and the internal consistencies of the CT subscales measured with Cronbach’s alpha were acceptable, suggesting some internal consistency.

We found correlations between the countertransference subscales and the alliance measures to be significant in two different non-overlapping perspectives: the working alliance reported by the therapists that showed meaningful correlations with all the subscales as well as the working alliance reported by the patients that showed a correlation with the confident subscale. The link between the therapists’ feelings and the therapist–patient bond or working relationship is of particular interest in future research when investigating rupture and repair of the alliance. The quality of the working alliance has been shown to be more predictive of patient outcome rather than the type of intervention [55], and correlations between therapists’ emotional response and alliance measures could therefore be important for clinical purpose.

Countertransference was originally described as a phenomenon in psychodynamic theory. Recent empirical studies suggest that countertransference is not only associated with one theoretical orientation but is a universal phenomenon in human encounters [56,57,58]. Our findings suggests that the Feeling-Word Checklist—28 captures countertransference feelings and can provide a method for collecting information about the treatment process and is appropriate for further research in adolescent psychotherapy.

Overall, the present study is an attempt to further explore the clinical implications of countertransference feelings. More research is needed to address how CT feelings influence the psychotherapy process, the working alliance, and patient outcome in different treatment modalities. Despite the limitations, we believe that the present study contributes to the preexisting literature and provides motivation for further investigations. More research on adolescent patients is necessary, and we hope the present study can encourage more empirical work in this direction, preferably with larger sample sizes and quality assured methods.

## Figures and Tables

**Table 1:** Pre-treatment characteristics of depressed adolescents included in the First Experimental Study Transference Work—In Teenagers.

	Transference Group (*n* = 36)		Non-Transference Group (*n* = 26)	
	n = 36	%	*n* = 26	%
Female gender	31	86.1%	20	76.9%
Depressive disorder	36	100%	26	100%
Comorbid diagnoses				
*Personality Disorder (PD criteria measured with SIDP-IV)*	18	50%	11	42.3%
M.I.N.I				
*Recurrent depression*	11	30.6%	8	30.8%
*Suicide risk (moderate to high)*	8	22%	4	15.4
*Eating disorder*	1	2.8%	1	3.8%
*PTSD*	1	2.8%	0	0%
*General anxiety*	12	33.3%	5	19.2%
*Social phobia*	7	19.4%	2	7.7%
*Panic disorder*	7	19.4%	3	11.5%
*Agoraphobia*	5	14%	2	7.7%
	Mean (*n* = 36)	(*SD*)	Mean (*n* = 26)	(*SD*)
Age	17.3	*0.74*	17.3	*0.75*
MADRS ^1^*	58.3	*6.8*	58.4	*5.0*
GAF *	24.2	*7.6*	24.3	*7.4*
BDI	28.3	*9.9*	28.1	*7.9*

^1^* Based on therapist’s and evaluator’s ratings; * based on evaluators rating. Abbreviations: PD, personality disorders; SIDP-IV, Structured Interview for DSM-IV Personality; MADRS, Montgomery–Åsberg Depression Rating Scale; GAF, General Assessment of Functioning; BDI, Beck Depression Inventory.

**Table 2:** The Feeling-Word Checklist—28: pattern matrix obtained via promax rotation showing the unique relationships between each factor and each observed item.

	Inadequate	Confident	Motherly	Disengaged
Tense	**0.848**	0.148	−0.150	−0.005
Overwhelmed	**0.801**	0.289	0.210	−0.107
Thunderstruck	**0.736**	0.078	−0.091	−0.023
Nervous	**0.656**	0.152	−0.273	−0.100
Ashamed	**0.449**	−0.038	0.159	0.075
Happy	0.060	**0.900**	−0.125	0.106
Playful	0.099	**0.799**	−0.083	−0.003
Satisfied	−0.127	**0.712**	0.052	0.077
Enthusiastic	0.067	**0.613**	0.144	−0.181
Energetic	0.161	**0.466**	0.217	−0.088
Motherly	0.278	−0.348	**0.847**	−0.091
Affectionate	0.122	0.002	**0.830**	−0.084
Warm	−0.051	0.283	**0.595**	0.026
Touched	0.017	0.027	**0.554**	−0.305
Important	−0.147	0.237	**0.542**	0.112
Indifferent	−0.129	−0.140	−0.089	**0.792**
Neutral	−0.169	0.133	−0.101	**0.760**
Cold	0.077	−0.037	−0.210	**0.672**
Prudent	0.201	0.283	0.090	**0.618**
Tired of	0.322	−0.347	0.043	**0.386**

Note: Loadings > 0.35 are bolded in the table. Eight items were removed, as they did not load strongly enough or loaded strongly on two components. Items removed: dominating, open, powerful, relaxed, irritated, calm, surprised, and free.

**Table 3:** Pearson correlations coefficients between the four CT subscales in the FWC-28, both on aggregated data and data from each session as separate measurements.

	Inadequate	Confident	Motherly	Disengaged
*All data (n = 174)*				
Inadequate	1	−0.215 *	−0.141	0.222 *
Confident	−0.215 **	1	0.493 **	−0.126
Motherly	−0.141	0.493 **	1	−0.085
Disengaged	0.222 *	−0.126	−0.085	1
*Aggregated data (n = 62)*				
Inadequate	1	−0.061	−0.121	0.265 *
Confident	−0.061	1	0.511 *	−0.068
Motherly	−0.121	0.511 **	1	−0.140
Disengaged	0.265 *	−0.068	−0.140	1

** Correlation is significant at the 0.01 level (2-tailed). * Correlation is significant at the 0.05 level (2-tailed).

**Table 4:** Range and mean of CT subscales from all sessions.

	*n*	Minimum	Maximum	Mean	Std. Deviation
Inadequate	174	0.00	2	0.54	0.45
Confident	174	0.00	2.8	1.34	0.57
Motherly	174	0.00	3	1.65	0.57
Disengaged	174	0.00	2.6	0.66	0.44

*n* = 174: All data collected from sessions 3, 12, 20, and 28.

**Table 5:** Pearson correlation between the CT subscales and alliance measures.

Alliance	Inadequate	Confident	Motherly	Disengaged
*All data*				
WAI-T all (n = 172)	−0.322 **	0.502 **	0.435 **	−0.286 **
VAS all (n = 168)	−0.287 **	0.457 **	0.460 **	−0.405 **
WAI-P all (n = 159)	0.070	0.186 *	0.155	−0.140
*Aggregated data *				
WAI-T ag (n = 62)	−0.352 **	0.598 **	0.594 **	−0.347 **
VAS ag (n = 62)	−0.254 *	0.584 **	0.580 **	−0.426 **
WAI-P ag (n = 58)	0.152	0.345 **	0.148	−0.037

Note: We included data on WAI and VAS scores from sessions with the 62 patients across the four measurement points as separate measurements and the average scores of the four measurement points. WAI-P, WAI-T, and VAS scores were available for 159, 172, and 168 sessions, respectively, out of the total of 174 possible correlations with CT subscales. ** Correlation is significant at the 0.01 level (2-tailed). * Correlation is significant at the 0.05 level (2-tailed).

## Data Availability

The data used in this article are available in an anonymized version by request from the first author.
